# Reliability of self-report measures of correlates of obesity-related behaviours in Hong Kong adolescents for the iHealt(H) and IPEN adolescent studies

**DOI:** 10.1186/s13690-017-0209-5

**Published:** 2017-09-25

**Authors:** Ester Cerin, Cindy H. P. Sit, Anthony Barnett, Wendy Y. J. Huang, Gemma Y. Gao, Stephen H. S. Wong, James F. Sallis

**Affiliations:** 1 School of Public Health, The University of Hong Kong, Hong Kong, China; 2 Institute for Health and Ageing, Australian Catholic University, Level 6, 215 Spring Street, Melbourne, VIC 3000 Australia; 3 Department of Sports Science and Physical Education, The Chinese University of Hong Kong, Hong Kong, China; 4 Department of Physical Education, Hong Kong Baptist University, Hong Kong, China; 5 Division of Behavioral Medicine, University of California – San Diego, San Diego, CA USA

**Keywords:** Exercise, Diet, Eating, Sedentary, Ecological model, Chinese

## Abstract

**Background:**

This study examined the reliability of measures of correlates of dietary behaviours (DBs), physical activity (PA) and sedentary behaviour (SB) for Hong Kong adolescents.

**Method:**

Individual, social and environmental correlates of obesity-related behaviours were assessed twice, 15–27 days apart (average 20 days), via self-administered questionnaires. These questionnaire included measures of decisional balance, self-efficacy, enjoyment and social support related to intake of fruits, vegetables, high-fat foods and sugar-sweetened beverages, PA behaviour and SB. They also included measures of perceived barriers to PA, parental rules related to PA and SB, and environmental correlates of DB, PA and SB. The questionnaires were self-completed outside school hours. A sample of 119 12–17 year old Chinese-speaking secondary school students (60 girls; 59 boys) were recruited from four Hong Kong schools located in areas stratified by walkability and socio-economic status.

**Results:**

The test-retest reliability of the examined measures ranged from poor to excellent (ICC: 0.30–0.99). All measures of correlates of PA and SB had excellent or substantial test-retest reliability, with the exception of self-efficacy for reducing SB (ICC: 0.59). Four of 18 measures of DBs showed moderate, and two poor (ICC < 0.41), test-retest reliability. Evidence of unidimensionality (Cronbach’s α ≥ 0.70) was found for 10 of 28 multi-item scales. The evidence for the remaining 18 was either questionable or poor.

**Conclusions:**

Most of the self-report measures of correlates of obesity-related behaviours used in the iHealt(H) study have acceptable test-retest reliability in Hong Kong adolescents. The factorial structure of several scales needs to be investigated in a larger sample.

**Electronic supplementary material:**

The online version of this article (doi:10.1186/s13690-017-0209-5) contains supplementary material, which is available to authorized users.

## Background

Adolescence is the most important period for predicting adult obesity [1]. It is, thus, imperative to focus on obesity prevention strategies in this age group by targeting relevant lifestyle behaviours including physical activity (PA), sedentary behaviour (SB) and dietary behaviours (DBs). PA is a well-established factor associated with better health and lower risk of obesity in young people [1–3]. SB has been shown to increase the risk of chronic non-communicable diseases in adults [4] and contribute to poor cardio-metabolic [5] health and obesity in youth [6, 7], independently of engagement in moderate-to-vigorous PA. Energy-dense dietary patterns typified by high fat intake, low intake of fruits/vegetables, and high free sugar intake (e.g., sugar-sweetened beverages) have been associated with higher levels of adiposity in youth [8]. As adolescence is the period during which PA shows a substantial (60–70%) decline [9], SB increases [10] and DBs are established or consolidated [11], improving eating, PA and SB in adolescents is a key global strategy for obesity prevention.

Although the prevalence of overweight and obesity in the general population is considerably lower in Chinese urban areas such as Hong Kong (39%) than in many developed countries (50–70%) [12, 13], the statistics on adolescents are worrying, with the prevalence of overweight/obesity progressively increasing in Hong Kong from 14% in 1997/98 to 19% in 2010/11 [14]. As to obesity-related behaviours (ORBs), more than 50% of Hong Kong adolescents were found to engage in insufficient amounts of PA and excessive SB [15–17]. Only 10% of adolescents reported consuming the recommended amounts of fruit and vegetables, <40% removed visible fat from meats, and more than 90% of youth consumed sugar-sweetened beverages [15]. To improve healthful behaviours among Hong Kong adolescents, it is important to identify modifiable factors promoting engagement in such behaviours [15].

Ecological models posit behaviours are the function of multiple levels of influence – namely, individual, social and physical-environmental - and their interactions [18]. Correlates of PA, SB and DBs in adolescents have been examined to varying degrees across one or two of the three levels of influence, with the vast majority of research in this area focussed on US adolescents [1, 19–24]. Studies on Hong Kong adolescents and adolescents from other Chinese metropolises are rare [15]. Studies assessing the interactive effects of various levels of influence are also rare in any population of adolescents [25], although this is one of the fundamental premises of ecological models of health behaviours [26].

An inspection of the literature on individual-level factors contributing to ORBs in adolescents reveals strong support for adolescents’ domain-specific self-efficacy being positively related to PA and healthy DBs, and negatively related to SB. In contrast, the evidence for many other individual-level factors, such as attitudes towards a behaviour or perceived barriers to engaging in a particular behaviour, is inconsistent or lacking [19, 20, 24]. Social factors including various sources of social support, parental modelling and parenting style/practices have emerged as significant influences on all three sets of ORBs [23–25, 27]. Yet, a recent review concluded that school (PA facilities) and neighbourhood environmental factors (walkability, PA facilities and traffic and crime safety) [28] may be stronger determinants of PA in adolescents than social factors [21]. With regards to SB, limited evidence suggests that social and environmental factors may be equally important [29]. The evidence related to DBs points at the importance of social factors and the home environment (parental modelling, parenting styles/rules, availability of foods), while evidence about the school and neighbourhood environments is weaker or lacking [21, 30, 31]. Research on correlates of PA in Hong Kong adolescents is limited and inconsistent [32], and evidence on correlates of DBs and SB is even more limited [33]. Clearly, there is much to be learnt with respect to correlates and determinants of adolescents’ PA, SB and DB globally and even more so within a Chinese urban context (e.g., Hong Kong).

Adopting an ecological framework, the iHealt(H) [international Healthy environments and active living in teenagers – (Hong Kong)] study aims to investigate the potential influence of individual, social and environmental facilitators and barriers to engagement in ORBs (i.e., PA, SB and DBs) in Hong Kong adolescents [34]. The iHealt(H) protocol mirrors that of the TEAN (Teen Environment And Neighborhood; http://sallis.ucsd.edu/measure_tean.html) study conducted in the U.S. in 2007–2011, allowing inter-country comparison. The PA and SB components of iHealt(H) also represent the Hong Kong arm of the multi-country IPEN Adolescent (International Physical activity and the Environment Network Adolescent; http://wwww.ipenproject.org/IPEN_adolescent _html) project aiming to accurately estimate associations of the environment with PA and SB in adolescents by maximising the variability of environmental exposure [35].

Given that self-report measures of individual, social and environmental correlates of ORBs for the TEAN and IPEN Adolescent studies were originally developed for U.S. adolescents, it is necessary to establish the measurement properties of these scales translated and adapted for Chinese-speaking Hong Kong adolescents. Hong Kong represents the IPEN Adolescent study site that differs the most from the place where the measures originated (U.S.A). Consequently, it is particularly important to assess the reliability (test-retest and internal consistency) of these measures in Hong Kong.

## Methods

### Participants and procedures

Hong Kong adolescents were recruited from local secondary schools. Using random stratified sampling, four schools were selected based on the level of walkability and socio-economic status of their census administrative area. Area walkability was defined using Geographic Information Systems data on dwelling density, street intersection density and land use mix [35], while area socio-economic status was defined using Census data on median household income. All four schools that were contacted consented to participate. They were located in one of the following area types: high walkable, high socio-economic status; high walkable, low socio-economic status; low walkable, high socio-economic status; and low walkable, high socio-economic status. We recruited participants from high and low walkable and high and low socio-economic status areas to maximize the variability in social and environmental correlates of PA, SB and DBs [21, 30, 31, 35].

The study aimed to recruit ~120 secondary-school students aged 12 to 17 years, approximately balanced by gender and age groups (12–13, 14–15 and 16–17 years). Power calculations indicated that this would allow detection of an intra-class correlation coefficient (ICC, a measure of test-retest reliability) of 0.40 (corresponding to minimally acceptable value) in each gender group (*n* = 60) with 90% power while adopting a probability level of 0.05 [36]. Eligibility criteria were being a 12–17 year old secondary school student, being able to read and write in Chinese, residing in a pre-selected area for at least 6 months, and not suffering from a disability/illness impeding engagement in moderate-intensity PA and/or from food allergies. Students were screened for eligibility. Parental consent and student’s assent were obtained prior to participation. The study was approved by the Human research Ethics Committee for Non-Clinical Faculties of the University of Hong Kong (# EA351010).

The sample consisted of 119 participants (59 boys; 60 girls; mean age: 15.2 years; response rate: 56%) recruited from the selected schools with assistance of the school staff. In April-May 2012, students self-completed the surveys in their free time and returned the completed surveys to their school. They were given the second survey 14–18 days after the date of completion noted in the first survey. The average time of completion between the first and second surveys was 20 days (range 15 to 27 days). This test-retest time interval corresponded to that used in the test-retest reliability assessment of the original measures [37]. Participants received a HK$50 voucher upon successful completion of both surveys as a token of appreciation for their time and commitment to the study.

### Measures

The iHealt(H) study uses self-report measures of individual, social and environmental correlates of adolescents’ ORBs reported by adolescents and their parents/caregivers, as well as objective measures of environmental correlates of adolescents’ ORBs. The present reliability study focused on self-report measures of correlates of ORBs as reported by adolescents. They included measures of decisional balance, self-efficacy, enjoyment and social support related to intake of fruits, vegetables, high-fat foods and sugar-sweetened beverages, PA behaviour and SB (Table 1). They also included measures of perceived barriers to engaging in PA, parental rules related to PA and SB, and environmental correlates of DBs, PA and SB.

**Table 1:** Characteristics of measures of correlates of obesity-related behaviours included in the current study

Measures	Description	Source and adaptations	Test-retest reliability^a^	Cronbach’s α
Dietary behaviour				
*Individual correlates*				
Decisional balance for eating fruits and vegetables	5 items about ‘Pros’ and 4 items about ‘Cons’ rated on 4-point Likert scale	Adapted for TEAN study from Hagler et al. [40]	Pros: 0.87 [40] Cons: 0.74 [40]	Pros: 0.78 [40] Cons: 0.72 [40]
Decisional balance for eating high-fat foods	4 items about ‘Pros’ and 3 items about ‘Cons’ rated on 4-point Likert scale	Adapted for TEAN study from Hagler et al. [40]	Pros: 0.85 [40] Cons: 0.71 [40]	Pros: 0.64 [40] Cons: 0.79 [40]
Decisional balance for drinking sugar-sweetened beverages	3 items about ‘Pros’ rated on 4-point Likert scale	TEAN study	Unknown	Unknown
Self-efficacy for eating fruits and vegetables	5 items rated on a 5-point scale ranging from ‘I’m sure I can’t’ to ‘I’m sure I can’	Adapted for TEAN study from Hagler et al. [40]	0.87 [40]	0.77 [40]
Self-efficacy for eating low-fat foods	8 items rated on a 5-point scale ranging from ‘I’m sure I can’t’ to ‘I’m sure I can’	Included in TEAN study from Hagler et al. [40]	0.93 [40]	0.90 [40]
Self-efficacy for reducing sugar-sweetened beverage intake	2 items rated on a 5-point scale ranging from ‘I’m sure I can’t’ to ‘I’m sure I can’	TEAN study	Unknown	Unknown
Enjoyment of fruits and vegetables	Single item rated on 5-point Likert scale	TEAN study	Unknown	n/a
Enjoyment of high-fat foods	Single item rated on 5-point Likert scale	TEAN study	Unknown	n/a
Enjoyment of sugar-sweetened beverages	Single item rated on 5-point Likert scale	TEAN study	Unknown	n/a
*Social correlates*				
Social support for eating fruits and vegetables	3 items about ‘support from adults’ and 3 items about ‘support from peers’ rated on a 4-point Likert scale	Adapted for TEAN study from Hagler et al. [40]	Adults: 0.79 [40] Peers: 0.75 [40]	Adults: 0.74 [40] Peers: 0.74 [40]
Social support for eating high-fat foods	3 items about ‘support from adults’ and 3 items about ‘support from peers’ rated on a 4-point Likert scale	Adapted for TEAN study from Hagler et al. [40]	Adults: 0.93 [40] Peers: 0.77 [40]	Adults: 0.77 [40] Peers: 0.80 [40]
Social support for drinking sugar-sweetened beverages	3 items about ‘support from adults’ and 3 items about ‘support from peers’ rated on a 4-point Likert scale	TEAN study	Unknown	Unknown
*Environmental correlates*				
School food environment	4 dichotomous items (‘Yes’, ‘No’), one assessing healthy and 3 unhealthy school practices/policies	Active Where study [38]	Kappa range: 0.57–0.77 [38]	n/a
Physical activity behaviour				
*Individual correlates*				
Perceived barriers to active transport (cycling or walking) to/from school	19 items rated on a 4-point Likert scale	17 items from the Active Where study [38] and 2 items added by expert panel: ‘being tired’ and ‘having a tight schedule (no time)’	Original 17 items: 0.38–0.77 [38]	11-item version: 0.80 [41]
Perceived barriers to active transport to/from closest park	17 items rated on a 4-point Likert scale	Active Where study [38]	0.32–0.78 [38]	Unknown
Perceived barriers to active transport in the neighbourhood	9 items rated on a 4-point Likert scale	Active Where study [38]	0.35–0.63 [38]	Unknown
Decisional balance for engagement in physical activity	5 items about ‘Pros’ and 5 items about ‘Cons’ rated on 4-point Likert scale	Adapted for TEAN study from Norman et al. [42]	Pros: 0.74 [42] Cons: 0.86 [42]	Pros: 0.81 [42] Cons: 0.53 [42]
Self-efficacy for physical activity	6 items rated on a 5-point scale ranging from ‘I’m sure I can’t’ to ‘I’m sure I can’	Included in TEAN study from Norman et al. [42]	0.71 [42]	0.76 [42]
Enjoyment of physical activity	Single item rated on 5-point Likert scale	Included in TEAN study from Norman et al. [42]	0.43 [42]	n/a
*Social correlates*				
Social support for physical activity	3 items about ‘support from adults’ and 2 items about ‘support from peers’ rated on a 5-point frequency scale	Adapted for TEAN study from Norman et al. [42]	Adults: 0.78 [42] Peers: 0.68 [42]	Adults: 0.81 [42] Peers: 0.53 [42]
Parental rules about physical activity	14 dichotomous items (‘Yes’, ‘No’)	Active Where study [38]	% agreement: 50% - 78% [38]	n/a
*Environmental correlates*				
School physical activity equipment	6 dichotomous items (‘Yes’, ‘No’)	Active Where study [38]	% agreement: 77% - 86% [38]	n/a
Physical activity equipment at home	10 dichotomous items (‘Yes’, ‘No’) and 4-point frequency scales	Active Where study [38]	% agreement: 55%–67% Frequency scales: 0.49–0.75 [38]	n/a
Perceived neighbourhood traffic safety	6 items rated on a 4-point Likert scale	Neighbourhood Environment Walkability Scale – Youth [43]	Items: 0.41–0.57 [38] Scale: 0.67 [43]	0.81 [43]
Perceived neighbourhood crime safety	8 items rated on a 4-point Likert scale	Neighbourhood Environment Walkability Scale – Youth [43]	Items: 0.34–0.74 [38] Scale: 0.73 [43]	0.87 [43]
Physical activity friendly school policy	2 items rated on a 5-point frequency scale	Active Where study [38]	0.27–0.57 [38]	n/a
Sedentary behaviour				
*Individual correlates*				
Decisional balance for engagement in sedentary behaviour	6 items about ‘Pros’ and 6 items about ‘Cons’ rated on 4-point Likert scale	Adapted for TEAN study from Norman et al. [42]	Pros: 0.30 [42] Cons: 0.59 [42]	Pros: 0.61 [42] Cons: 0.58 [42]
Self-efficacy for reducing sedentary behaviour	7 items rated on a 5-point scale ranging from ‘I’m sure I can’t’ to ‘I’m sure I can’	Included in TEAN study from Norman et al. [42]	0.80 [42]	0.90 [42]
Enjoyment of sedentary behaviour	Single item rated on 5-point Likert scale	Included in TEAN study from Norman et al. [42]	0.29 [42]	n/a
*Social correlates*				
Social support for sedentary behaviour	Single item about ‘support from adults’ and 2 items about ‘support from peers’ rated on a 5-point frequency scale	Adapted for TEAN study from Norman et al. [42]	Adults: 0.93 [42] Peers: 0.77 [42]	Adults: n/a Peers: 0.58 [42]
Parental rules about sedentary behaviour	3 dichotomous items (‘Yes’, ‘No’)	Adapted for TEAN study from Salmon et al. [44]	% agreement: 71% - 90% [44]	n/a
*Environmental correlates*				
Screen media in bedroom	6 dichotomous items (‘Yes’, ‘No’)	Adapted from continuous items in Active Where study [38]	0.36–0.79 [38]	n/a
Personal electronics	4 dichotomous items (‘Yes’, ‘No’)	Adapted from continuous items in Active Where study [38]	0.38–0.76 [38]	n/a

^a^Values represent estimates of intra-class correlation (ICC) unless otherwise stated

All measures were based on those used in the Active Where [38] and TEAN studies, which employed or adapted extant validated instruments and, if appropriate instruments were not available, constructed and validated new questionnaire items suitable for U.S. adolescents (see Table 1). In the present study, a three-member bilingual (Chinese-English) panel of experts reviewed all selected measures and, when necessary, adapted extant items or added new items to capture important aspects of correlates of ORBs relevant to Chinese adolescents and/or Hong Kong. The measures were then translated from English to Chinese using traditional Chinese characters common in Hong Kong and Taiwan. They were then back-translated in English following World Health Organization guidelines [39]. A panel of four bilingual experts in the development and cross-cultural adaptation of health-related questionnaires reviewed the translations and iteratively resolved any discrepancies between the original and back-translated versions of the measures. The final Chinese working versions of the measures were pilot tested on five university and 10 secondary-school students for clarity. Table 1 provides a list of all the measures used in this study, including their source and, when available, psychometric characteristics (test-retest reliability and internal consistency) of the original versions [34, 37, 38, 40–44]. Data on test-retest reliability of the original versions were available for most measures with the exception of those related to sugar-sweetened beverage intake and enjoyment of various foods. Data on internal consistency were unavailable for 6 of the 28 original multi-item measures representing scales measuring a latent construct (Table 1). The measures used in this study (English translation) are available online (see Additional file 1).

#### Data analyses

Descriptive statistics (means and standard deviations) were computed for each measure for the whole sample and by adolescent gender. As measures represented scores on a scale (i.e., they yielded continuous data), test-retest reliabilities were established by computing two-way mixed effects ICCs. They were computed for the whole sample and by gender because gender differences in test-reliability were observed in relation to measures of PA and SB [34]. Based on previously proposed criteria, values below 0.40 were classified as poor, 0.41 to 0.60 as moderate, 0.61 to 0.80 as substantial and over 0.80 as excellent test-retest reliability [45]. Item-by-item test-retest reliabilities were not assessed because most measures had been validated in previous samples of adolescents and, within the context of the iHealt(H) and IPEN Adolescent studies, our main interest was in the performance of the total scores on the scales rather than individual items. Differences in mean values between the first and second assessments were tested using *t*-tests for dependent samples. Cronbach’s α was used to estimate the internal consistency (i.e., unidimensionality) of measures supposed to represent a unidimensional construct (e.g., self-efficacy or social support). Cronbach’s α values ≥0.70 were considered as providing sufficient evidence of unidimensionality, 0.60–0.70 as providing questionable evidence, and 0.50–0.60 as providing poor evidence [45]. Cronbach’s α values smaller than 0.50 were considered unacceptable. Multi-item measures consisting of checklists of equipment, rules and policies were treated as indices (rather than scales gauging unidimensional latent constructs) and, hence, their internal consistency was not assessed [45]. Between-gender differences in test-retest reliability and internal consistency estimates were tested using bootstrap methods, whereby 95% bootstrapped confidence intervals of differences between ICCs excluding 0 were considered statistically significant at a probability level of 0.05. All analyses were conducted in R.

## Results

Table 2 summarises the results of this study for the whole sample and by adolescent gender. On average, Hong Kong adolescents reported higher levels of pros than cons associated with healthful, obesity-preventing behaviours (fruits and vegetables intake and PA) (see Decisional balance – pros and cons scales in Table 2). The differences between average levels of perceived pros and cons associated with obesity-promoting behaviours (high-fat foods intake and SB) were small. The highest average score on self-efficacy measures was observed for reduction of sugar-sweetened beverages, and the lowest for engagement in PA. The highest levels of enjoyment were reported for SB and fruits and vegetable intake, and the lowest for sugar-sweetened beverage consumption. In general, participants exhibited low levels of perceived barriers to engaging in PA, with barriers to active transport to/from school being the most prominent. Participants received more social support for engagement in obesity-preventing behaviours from adults than peers, with the exception of SB. A higher percentage of parental rules about PA (average of seven out of 14 rules) than SB (average of one out of three rules) was endorsed. Participants reported an average of approximately three of four assessed features of the school environment promoting unhealthy DBs and gave an average score of 2.5 out of 4 on school policies promoting PA. In general, the neighbourhood environment was perceived as being safe, with safety from crime rating higher than traffic safety. No significant gender differences were found in any of the examined correlates of ORBs.

**Table 2:** Descriptive statistics and reliability of self-report measures of correlates of obesity-related behaviours for the iHealt(H) and IPEN Adolescent studies in Hong Kong adolescents

	Overall sample (*N* = 119)			Boys (*n* = 59)			Girls (*n* = 60)		
Measure [theoretical range: number of items]	Mean^a^ (SD)	ICC (95% CI)	Cronbach α^a^	Mean^a^ (SD)	ICC (95% CI)	Cronbach α^a^	Mean^a^ (SD)	ICC (95% CI)	Cronbach α^a^
Dietary behaviour									
Individual correlates									
Pros for eating fruits and vegetables [1–4 : 5]	3.0 (0.6)	0.86 (0.74, 0.92)	0.75	2.9 (0.6)	0.82 (0.68, 0.90)	0.78	3.1 (0.5)	0.87 (0.72, 0.94)	0.70
Cons for eating fruits and vegetables [1–4 : 4]	1.7 (0.5)	0.72 (0.62, 0.83)	0.64	1.7 (0.6)	0.71 (0.57, 0.86)	0.67	1.7 (0.5)	0.72 (0.58, 0.87)	0.61
Pros for eating high-fat foods [1–4 : 4]	2.1 (0.6)	0.69 (0.59, 0.79)	0.69	2.1 (0.7)	0.67 (0.53, 0.81)	0.67	2.0 (0.6)	0.71 (0.56, 0.87)	0.72
Cons for eating high-fat foods [1–4 : 3]	2.5 (0.6)	0.76 (0.67, 0.85)	0.59	2.4 (0.7)	0.78 (0.65, 0.93)	0.59	2.6 (0.6)	0.75 (0.62, 0.89)	0.58
Pros for drinking sugar-sweetened beverages [1–4 : 3]	2.4 (0.4)	0.68 (0.57, 0.79)	0.56	2.5 (0.4)	0.64 (0.52, 0.76)	0.60	2.4 (0.4)	0.70 (0.55, 0.88)	0.52
Self-efficacy for eating fruits and vegetables [1–5 : 5]	3.2 (0.9)	0.92 (0.90, 0.94)	0.83	3.0 (0.9)	0.88 (0.81, 0.93)	0.83	3.4 (0.8)	0.93 (0.75, 0.98)	0.82
Self-efficacy for eating low-fat foods [1–5 : 8]	3.0 (0.9)	0.81 (0.77, 0.86)	0.91	2.9 (0.9)	0.80 (0.66, 0.88)	0.91	3.1 (0.8)	0.82 (0.74, 0.88)	0.91
Self-efficacy for reducing sugar-sweetened beverage intake [1–5 : 2]	3.6 (1.0)	0.77 (0.68, 0.86)	0.69	3.5 (1.1)	0.72 (0.58, 0.87)	0.68	3.7 (1.0)	0.82 (0.76, 0.87)	0.70
Enjoyment of fruits and vegetables [1–5 : 1]	3.9 (0.9)	0.90 (0.84, 0.95)	n/a	3.8 (0.9)	0.88 (0.80, 0.94)	n/a	4.0 (0.8)	0.91 (0.83, 0.97)	n/a
Enjoyment of high-fat foods [1–5 : 1]	3.1 (1.0)	0.34 (0.16, 0.49)	n/a	3.3 (1.1)	0.30 (0.11, 0.54)	n/a	2.9 (1.2)	0.38 (0.12, 0.59)	n/a
Enjoyment of sugar-sweetened beverages [1–5 : 1]	3.0 (1.0)	0.40 (0.21, 0.54)	n/a	3.2 (1.0)	0.41 (0.23, 0.59)	n/a	2.9 (1.0)	0.39 (0.10, 0.58)	n/a
Social correlates									
Social support for eating fruits and vegetables from adults [1–4 : 3]	3.2 (1.0)	0.51 (0.34, 0.64)	0.53	3.0 (0.9)	0.54 (0.39, 0.68)	0.49	3.3 (1.0)	0.49 (0.34, 0.66)	0.57
Social support for eating fruits and vegetables from peers [1–4 : 3]	1.6 (1.0)	0.44 (0.35, 0.51)	0.65	1.5 (1.0)	0.41 (0.24, 0.58)	0.64	1.7 (0.9)	0.46 (0.30, 0.63)	0.66
Social support for eating less high-fat foods from adults [1–4 : 3]	2.4 (1.2)	0.65 (0.56, 0.73)	0.54	2.1 (1.0)	0.64 (0.51, 0.77)	0.57	2.7 (1.2)	0.66 (0.54, 0.79)	0.53
Social support for eating less high-fat foods from peers [1–4 : 3]	1.3 (0.7)	0.56 (0.45, 0.65)	0.55	1.2 (0.4)	0.56 (0.41, 0.70)	0.60	1.4 (0.8)	0.56 (0.40, 0.70)	0.52
Social support for drinking sugar-sweetened beverages from adults [1–4 : 3]	2.0 (0.6)	0.64 (0.51, 0.74)	0.64	2.0 (0.6)	0.62 (0.50, 0.74)	0.63	1.9 (0.5)	0.65 (0.52, 0.78)	0.66
Social support for drinking sugar-sweetened beverages from peers [1–4 : 3]	2.3 (0.5)	0.67 (0.56, 0.77)	0.68	2.3 (0.5)	0.62 (0.51, 0.73)	0.65	2.2 (0.5)	0.70 (0.54, 0.89)	0.70
Environmental correlates									
School food environment (unhealthy) [0–4 : 4]	2.9 (0.9)	0.56 (0.45, 0.65)	n/a	2.9 (0.9)	0.52 (0.37, 0.66)	n/a	2.9 (0.9)	0.60 (0.47, 0.73)	n/a
Physical activity (PA)									
Individual correlates									
Perceived barriers to active transport to/from school [1–4 : 19]	2.2 (0.6)	0.76 (0.66, 0.85)	0.91	2.2 (0.7)	0.75 (0.62, 0.88)	0.91	2.3 (0.6)	0.77 (0.63, 0.88)	0.90
Perceived barriers to active transport to/from closest park [1–4: 17]	1.9 (0.7)	0.61 (0.48, 0.73)	0.92	1.9 (0.7)	0.65 (0.50, 0.76)	0.93	1.9 (0.7)	0.57 (0.40, 0.71)	0.91
Perceived barriers to PA in the neighbourhood [1–4 : 9]	1.7 (0.6)	0.67 (0.56, 0.76)	0.83	1.7 (0.7)	0.60 (0.43, 0.75)	0.85	1.7 (0.6)	0.73 (0.60, 0.87)	0.82
Pros for engagement in PA [1–4 : 5]	3.2 (0.6)	0.80 (0.71, 0.90)	0.78	3.3 (0.6)	0.78 (0.64, 0.87)	0.78	3.2 (0.6)	0.81 (0.74, 0.86)	0.77
Cons for engagement in PA [1–4 : 5]	1.9 (0.5)	0.68 (0.57, 0.76)	0.61	1.8 (0.5)	0.65 (0.53, 0.74)	0.62	1.9 (0.5)	0.69 (0.46, 0.83)	0.61
Self-efficacy for PA [1–5 : 6]	2.7 (1.0)	0.73 (0.63, 0.85)	0.88	2.9 (1.0)	0.72 (0.58, 0.90)	0.87	2.6 (0.9)	0.73 (0.62, 0.86)	0.87
Enjoyment of PA [1–5 : 1]	3.7 (1.0)	0.65 (0.53, 0.75)	n/a	4.0 (1.0)	0.63 (0.47, 0.73)	n/a	3.6 (1.0)	0.66 (0.51, 0.75)	n/a
Social correlates									
Social support for PA from adults [0–4 : 3]	1.5 (0.9)	0.79 (0.68, 0.88)	0.68	1.4 (1.0)	0.73 (0.59, 0.89)	0.66	1.5 (0.9)	0.81 (0.72, 0.88)	0.71
Social support for PA from peers [0–4 : 2]	1.1 (1.0)	0.74 (0.62, 0.82)	0.69	1.1 (1.1)	0.69 (0.57, 0.77)	0.72	1.2 (1.0)	0.78 (0.62, 0.89)	0.68
Parental rules about PA [0–14 : 14]	7.0 (3.5)	0.75 (0.66, 0.86)	n/a	6.4 (3.6)	0.73 (0.49, 0.87)	n/a	7.5 (3.3)	0.76 (0.68, 0.84)	n/a
Environmental correlates									
School physical activity equipment [0–6 : 6]	4.6 (1.1)	0.74 (0.60, 0.89)	n/a	4.6 (1.1)	0.75 (0.59, 0.90)	n/a	4.5 (1.1)	0.73 (0.58, 0.87)	n/a
Physical activity equipment at home [0–10 : 10]	5.0 (2.4)	0.98 (0.95, 0.99)	n/a	5.1 (2.5)	0.89 (0.82, 0.93)	n/a	4.9 (2.4)	0.99* (0.98, 1.00)	n/a
Perceived neighbourhood traffic safety [1–4 : 6]	3.0 (0.4)	0.81 (0.71, 0.86)	0.59	3.1 (0.4)	0.80 (0.66, 0.88)	0.65	3.0 (0.4)	0.81 (0.68, 0.89)	0.53
Perceived neighbourhood crime safety [1–4 : 8]	3.2 (0.5)	0.75 (0.68, 0.83)	0.82	3.4 (0.6)	0.78 (0.65, 0.83)	0.81	3.1 (0.5)	0.73 (0.61, 0.86)	0.82
Physical activity friendly school policy [0–4 : 2]	2.5 (0.8)	0.70 (0.60, 0.78)	n/a	2.5 (0.9)	0.65 (0.53, 0.78)	n/a	2.6 (0.8)	0.78 (0.62, 0.89)	n/a
Sedentary behaviour (SB)									
Individual correlates									
Pros for engagement in SB [1–4 : 6]	2.6 (0.5)	0.71 (0.61, 0.82)	0.57	2.7 (0.5)	0.73 (0.60, 0.88)	0.59	2.6 (0.5)	0.70 (0.47, 0.84)	0.56
Cons for engagement in SB [1–4 : 6]	2.5 (0.5)	0.66 (0.55, 0.76)	0.53	2.4 (0.5)	0.61 (0.49, 0.73)	0.52	2.6 (0.4)	0.69 (0.45, 0.85)	0.51
Self-efficacy for reducing SB [1–5 : 7]	3.1 (0.8)	0.59 (0.48. 0.68)	0.76	3.0 (0.9)	0.60 (0.43, 0.75)	0.78	3.2 (0.7)	0.58 (0.44, 0.72)	0.74
Enjoyment of SB [1–5 : 1]	3.9 (0.9)	0.77 (0.67, 0.86)	n/a	4.0 (0.9)	0.75 (0.62, 0.89)	n/a	3.9 (0.9)	0.80 (0.67, 0.88)	n/a
Social correlates									
Social support for SB from adults [0–4 : 1]	2.2 (1.2)	0.68 (0.56, 0.80)	n/a	2.1 (1.2)	0.66 (0.53, 0.80)	n/a	2.4 (1.2)	0.69 (0.44, 0.86)	n/a
Social support for SB from peers [0–4 : 2]	1.9 (0.8)	0.72 (0.62, 0.83)	0.55	1.9 (0.8)	0.68 (0.54, 0.82)	0.56	1.8 (0.8)	0.76 (0.62,0.87)	0.58
Parental rules about SB [0–3 : 3]	1.0 (1.1)	0.80 (0.72, 0.89)	n/a	1.0 (1.1)	0.81 (0.67, 0.90)	n/a	1.0 (1.0)	0.80 (0.70, 0.87)	n/a
Environmental correlates									
Screen media in bedroom [0–6: 6]	1.9 (1.5)	0.96 (0.92, 0.99)	n/a	2.0 (1.6)	0.92 (0.85, 0.96)	n/a	1.8 (1.4)	0.99* (0.98, 1.00)	n/a
Personal electronics [0–4 : 4]	2.7 (0.9)	0.78 (0.68, 0.87)	n/a	2.7 (1.0)	0.77 (0.64, 0.87)	n/a	2.7 90.8)	0.78 (0.66, 0.89)	n/a

^a^Differences between means and internal consistencies (Cronbach’s α) at first and second assessments not statistically significant (all *p*s > 0.13). Thus, only means, standard deviations and Cronbach’s α values for data collected at the first assessment are reported

*p < .05

The test-retest reliability of the measures included in this study ranged from poor to excellent. The latter included pros, self-efficacy and enjoyment related to eating fruits and vegetables; self-efficacy for eating low-fat foods; pros for engagement in PA; PA equipment at home; perceived neighbourhood traffic safety; parental rules about SB; and screen media in the bedroom. All remaining measures of correlates of PA and SB had substantial test-retest reliability, with the exception of self-efficacy for reducing SB. In contrast, several measures of DBs showed poor-to-moderate test-retest reliability. Among these, the worst performing measures were enjoyment of high-fat foods and sugar-sweetened beverages with unacceptable test-retest reliability (Table 2). Only two significant between-gender differences in ICCs were found, one for the measure of PA equipment at home, the other for screen media in the bedroom, whereby girls showed better test-retest reliability than boys.

Evidence of sufficient internal consistency (Cronbach’s α ≥ 0.70), and hence support for their unidimensionality, was found for 10 out of 28 multi-item scales (Table 2). The internal consistencies of nine scales were deemed questionable (0.70 > Cronbach’s α ≥ 0.60) and those of the remaining nine poor (0.60 > Cronbach’s α ≥ 0.50). Most of these scales consisted of a small number of items (2 to 4). Pros and cons for engagement in SB, and perceived neighbourhood traffic safety were the only scales with more than four items showing poor internal consistency. No significant between-gender differences in internal consistency were found.

## Discussion

The iHealt(H) study represents the Chinese (Hong Kong) arm of the multi-country IPEN Adolescent study on individual, social, environmental and behavioural determinants of adolescents’ overweight/obesity [34]. The methodology used in the IPEN Adolescent study, including sampling methods and measures, mirrored that of the TEAN study conducted in the U.S. It was, thus, necessary to translate and, where necessary, adapt all relevant self-report measures from the TEAN study for use with Hong Kong Chinese-speaking adolescents. The main aim of this investigation was to examine the test-retest reliability and, where appropriate, internal consistency of the Chinese versions of 42 self-report measures of correlates of ORBs (DBs, PA and SB) used in the iHealt(H) study.

### Test-retest reliability

Acceptable levels of test-retest reliability were found for all measures with the exception of enjoyment of high-fat foods and sugar-sweetened beverages. The lower levels of repeatability for high-fat foods and sugar-sweetened beverages could be due to Hong Kong adolescents being more ambivalent towards these foods as compared to fruits and vegetables, and engagement in PA and SB. In fact, the average enjoyment score for high-fat and sugar-sweetened beverages observed in this study corresponded to the descriptor ‘neutral’. This is contrast to the other three behaviours about which participants expressed stronger, more definite opinions (i.e., they were rated as ‘enjoyable’). Studies have shown that test-retest reliability tends to be lower when the distribution of responses on a scale is centred around a neutral-response midpoint (indicating a degree of ‘uncertainty’) than above or below the midpoint [46].

Measures of pros and cons for engaging in DBs, PA and SB showed similar substantial-to-high levels of test-retest reliability, which were comparable to those found in U.S. samples of adolescents [40, 42]. In contrast, test-retest reliability tended to be higher for self-efficacy measures related to DBs than PA and SB (Table 2). Interestingly, this pattern of findings was also observed in validation studies of the original measures [40, 42]. The ability of adolescents to control or predict their food intake may be greater than their ability to control their engagement in PA and SB. Dietary intake in this age group is largely influenced by the home environment [21, 30, 31] and family habits, which are likely stable and, thus, predictable. Conversely, it has been suggested that PA and SB may be more affected by environmental and social factors [21, 29], including academic and other time commitments. As these factors are less controllable and more variable across time, they might negatively affect the stability of scores on PA/SB self-efficacy measures by influencing the actual behaviours in question.

Differences in test-retest reliability between DBs and PA/SB were also observed for measures of social support, with those related to PA/SB (average ICC: 0.73) generally outperforming their DBs counterparts (average ICC: 0.58). Again, these findings are consistent with previous validation studies [40, 42] and may be due to eating and drinking occurring in a greater variety of settings and contexts (home, school, food outlets, with others, alone, etc.) than the sedentary and PA behaviours described in measures of social support (i.e., participation in sports, watching TV and playing electronic games with others, walking/cycling to school or a friend’s house). Changes in participants’ perception of the amount of social support received from others may be a reflection of changes in settings and contexts within which certain behaviours are performed.

Measures of student-reported parental rules about PA and SB had, respectively, substantial and excellent test-retest reliability, which were within the range of those reported for the original measures [38, 44]. The latter also held true for measures of school PA equipment, personal electronics, perceived barriers to active transport to/from school and to/from the closest park [38] and perceived neighbourhood safety from crime [43], all of which showed substantial test-retest reliability. Finally, the translated/adapted measures of perceived barriers to active transport in the neighbourhood, PA-friendly school policy, PA at home, screen media in the bedroom [38] and neighbourhood traffic safety [43] displayed higher levels of test-retest reliability than their original English counterparts. Three of these measures consisted of lists of policies or equipment in the home, which might have been easier to report reliably given that the average size of a home in Hong Kong is a less than a third of the size of a home in the U.S.A. [47] and, thus, residents may be more aware of its content. Also, Hong Kong residents [48–50], engage in substantial amounts of active transport within and outside the neighbourhood. This may contribute to them being more reliable and accurate assessors of neighbourhood traffic safety and factors that act as barriers to active transport.

### Internal consistency

The measures assessing pros and cons of engaging in specific ORBs had poor (four measures) to acceptable (two measures) levels of internal consistency, which were, on average, slightly lower than those observed in U.S. adolescents [40, 42]. The relatively low levels of internal consistency may be in part due to the measures including a small number of items (three to six) and in part due to the measured constructs being multi-dimensional [51]. In fact, Kroll et al. [52] reported a seven-factor solution for a measure of decisional balance (including pros and cons for engaging in PA). Factors defining pros for engaging in PA were well-being, health, social contact and appearance, while cons included the factors of discomfort, exhaustion and costs. This suggests that the measures of pros and cons for engaging in ORBs that are being used in the iHealt(H) and IPEN Adolescent studies are likely to represent indices of various not-necessarily-related reasons for engagement in ORBs rather than scales of unidimensional constructs. Future studies will need to consider developing more comprehensive, multi-dimensional instruments of decisional balance related to ORBs for Chinese adolescents.

Similarly to what was reported in validation studies of the original measures [40, 42], all self-efficacy scales showed acceptable levels of internal consistency, with the exception of SB. While the self-efficacy scales related to DBs and PA consist of items referring to a single behaviour (being physically active or eating fruits and vegetables), the scale of self-efficacy for reducing SB includes items related to the reduction of a range of different behaviours, such as TV watching, internet use, listening to music and communicating with friends. It is possible that the perceived difficulty of reducing an activity varied by activity type. For example, adolescents might have been very confident in their ability to reduce the time they spent watching TV but less willing to reduce the time they spent talking with or texting friends. The measure of self-efficacy for reducing SB is likely to be multi-dimensional and its factorial structure will need to be thoroughly assessed in larger samples of adolescents.

The measures of social support from family and peers for engaging in ORBs used in this study showed poor-to-questionable levels of internal consistency, which were generally lower than those observed in U.S.A. adolescents [40, 42]. Yet, we need to note that our measures were very short and consisted of only two or three items, while those reported in published literature were three-to-five items long. As mentioned earlier, the number of items can have a substantial impact on the internal consistency of a scale [51]. It is also possible that the items included in the measures represented two somewhat independent dimensions of social support: one being engagement in a specific behaviour by the person providing social support (role model), the other being the provision of encouragement (to the adolescent) for engaging in a specific behaviour. In fact, post-hoc analyses excluding the items gauging role modelling resulted in a substantial increase in internal consistency (Cronbach’s α > 0.70).

Measures of perceived barriers to PA and neighbourhood crime safety had high levels of internal consistency, which is in line with previous studies [41, 43]. This was not the case for the six-item measure of perceived neighbourhood traffic safety comprising statements describing positive (e.g., ‘There are crosswalks and signals on busy streets’) as well as negative aspects of neighbourhood traffic (traffic speed and volume). This scale was originally taken from the NEWS-Y [43], which is the youth version of one of the most frequently used instruments of perceived attributes of the neighbourhood environment related to walking and PA [53, 54]. While the factorial structure of the NEWS-Y has yet to be established, several studies have examined the structure of the NEWS for adults and older adults [53–56]. Confirmatory factor analyses conducted in Australia [55] and Hong Kong [56] showed that the responses on five of the six items included in the perceived traffic safety scale examined in the present study were explained by three different weakly-to-moderately correlated latent factors: traffic safety/hazards, traffic speed/load and pedestrian infrastructure. In the USA, these items were found to be associated with two latent factors: traffic safety/hazards and infrastructure and safety for walking/cycling [56, 57]. These findings suggest that the current measure of perceived neighbourhood traffic safety is a multi-dimensional instrument similar to a checklist of traffic safety elements rather than a set of items gauging the same construct. Future studies on larger samples will need to assess its factorial structure.

### Strengths and limitations

This study had two main strengths. It is the first study to report the test-retest reliability and internal consistency of several self-report measures included in the on-going multi-country IPEN Adolescent study on determinants of overweight and obesity across the globe. Secondly, it systematically recruited adolescents residing in areas stratified by walkability and household income. This strategy is likely to have yielded more robust estimates of psychometric properties of the examined measures because it helped maximise the variability of the physical and social environmental factors influencing the ORBs of interest. Limitations of the study included the questionable representativeness of the sample given the 56% response rate; the inability to examine psychometric properties of the instruments by age groups due to the limited number of participants per age group; and the small number of items included in some of the measures. The last limitation was related to the need to follow a common multi-country study protocol that would allow data pooling and inter-country comparisons. In addition, studies of multiple behaviours and multi-level correlates need to use short scales to control the respondent burden. Future studies need to examine the factorial structure of the factor-analysable measures on larger representative samples of Chinese adolescents. They also need to examine the construct validity (e.g., association of the examined measures with adolescents’ ORBs) and potential floor and ceiling effects of the measures, which is within the scope of the iHealt(H) and IPEN Adolescent studies.

## Conclusions

This study suggests that, with a couple of exceptions, the Chinese self-report measures of individual, social and environmental correlates of ORBs used in the iHealt(H) study (the Chinese – Hong Kong arm of the multi-country IPEN Adolescent study) have acceptable levels of test-retest reliability that are, generally, comparable to those of the original English measures developed for U.S.A. adolescents. The level of internal consistency was acceptable for over a third of the measures that were assessed for this particular metric. Further work will need to establish the factorial structure of the measures showing signs of multi-dimensionality (i.e., low internal consistency) in appropriately large representative samples of Chinese adolescents. Similar work should be also undertaken in other populations of adolescents.

## Additional file

Additional file 1: Measures of correlates of obesity-related behaviours used in the iHealt(H) and IPEN Adolescent studies(DOCX 60 kb)

## Abbreviations

DB: Dietary behaviour
ICC: Intra-class correlation
iHealt(H): International Healthy environments and active living in teenagers – (Hong Kong)
IPEN Adolescent: International Physical Activity and the Environment Network Adolescent
NEWS-Y: Neighborhood Environment Walkability Scale for Youth
ORB: Obesity related behavior
PA: Physical activity
SB: Sedentary behavior
TEAN: Teen Environment and Neighborhood

## References

[1] Hills AP, King NE, Byrne NM (2007). Children, obesity and exercise: prevention, treatment and Management of Childhood and Adolescent Obesity.

[2] Riddoch C, Biddle S, Sallis JF, Cavill N (1998). Relationships between physical activity and health in young people. Young and active? Young people and health-enhancing physical activity – evidence and implications.

[3] Summerbell CD, Waters E, Edmunds LD, Kelly S, Brown T, Campbell KJ (2005). Interventions for preventing obesity in children. Cochrane Database Syst Rev.

[4] Owen N, Healy GN, Matthews CE, Dunstan DW (2010). Too much sitting: the population health science of sedentary behaviour. Exerc Sport Sci Rev.

[5] Sardinha LB, Andersen LB, Anderssen SA, Quitério AL, Ornelas R, Froberg K (2008). Objectively measured time spent sedentary is associated with insulin resistance independent of overall and central body fat in 9- to-10-year-old Portuguese children. Diabetes Care.

[6] Mitchell JA, Mattocks C, Ness AR, Leary SD, Pate RR, Dowda M (2009). Sedentary behaviour and obesity in a large cohort of children. Obesity.

[7] Steele RM, van Sluijs EMF, Cassidy A, Griffin SJ, Ekelund U (2009). Targeting sedentary time or moderate-and vigorous-intensity activity: independent relations with adiposity in a population based sample of 10-y-old British children. Am J Clin Nutr.

[8] Ambrosini GL, Johns DJ, Northstone K, Emmett PM, Jebb SA (2016). Free sugars and total fat are important characteristics of a dietary pattern associated with adiposity across childhood and adolescence. J Nutr.

[9] Dumith SC, Gigante DP, Dominigues MR, Kohl HW (2011). Physical activity change during adolescents: a systematic review and a pooled analysis. Int J Epidemiol.

[10] Mitchell JA, Pate RR, Beets MW, Nader PR (2013). Time spent in sedentary behavior and changes in childhood BMI: a longitudinal study from ages 9 to 15 years. Int J Obes.

[11] Lake AA, Adamson AJ, Craigie AM, Rugg-Gun AJ, Mathers JC (2009). Tracking of dietary intake and factors associated with dietary change for early adolescence to adulthood: the ASH30 study. Obes Facts.

[12] Department of Health, Hong Kong SAR. Health facts of Hong Kong, 2015 edition. Retrieved from http://www.dh.gov.hk/english/statistics/statistics_hs/files/Health_Statistics_pamphlet_E.pdf. Accessed 10 Apr 2017.

[13] World Health Organization. Global Health Observatory data repository. Overweight (body mass index >= 25) (age-standardized estimate). Data by country. 2015. http://apps.who.int/gho/data/node.main.A897A?lang=en. Accessed 10 04 2017.

[14] Student Health Service, Department of Health, Hong Kong SAR. Newsletters, Issue No. 57. 2012. http://www.studenthealth.gov.hk/english/newsletters/newsletter_57.html. Assessed 10 04 2017.

[15] Department of Health, HKSAR (2010). Action plan to promote healthy diet and physical activity participation in Hong Kong.

[16] Lam JWK, Sit CP, Cerin E (2010). Physical activity and sedentary behaviours in Hong Kong primary school children: prevalence and gender differences. Prev Med.

[17] Wong BY, Cerin E, Ho SY, Mak KK, Lo WS, Lam TH (2009). Adolescents’ physical activity: competition between perceived neighbourhood sport facilities and home media resources. Int J Pediatr Obes.

[18] Sallis JF, Cervero RB, Ascher W, Henderson KA, Kraft MK, Kerr J (2006). An ecological approach to creating active living communities. Annu Rev Public Health.

[19] Cerin E, Barnett A, Baranowski T (2009). Testing theories of dietary behavior change in youth using the mediating variable model with intervention programs. J Nutr Educ Behav.

[20] Craggs C, Corder K, van Sluijs EMF, Griffin SJ (2011). Determinants of change in physical activity in children and adolescents: a systematic review. Am J Prev Med.

[21] de Vet E, de Ridder DTD, de Wit JBF (2011). Environmental correlates of physical activity and dietary behaviours among young people: a systematic review of reviews. Obes Rev.

[22] Ding D, Sallis JF, Kerr J, Lee S, Rosenberg DE (2011). Neighborhood environment and physical activity among youth: a review. Am J Prev Med.

[23] Sallis JF, Prochaska JJ, Taylor WC (2000). A review of correlates of physical activity of children and adolescents. Med Sci Sports Exerc.

[24] van der Horst K, Chin A, Paw MJ, Twisk JW, van Mechelen W (2007). A brief review of correlates of physical activity and sedentariness in youth. Med Sci Sports Exerc.

[25] Carlson JA, Sallis JF, Kerr J, Conway TL, Cain K, Frank LD, Saelens BE (2014). Built environment characteristics and parent active transport are associated with active travel to school in youth age 12–15. Br J Sports Med.

[26] Sallis JF, Owen N, Glanz K, Rimer B, Viswanath V (2015). Ecological models of health behavior. Health behavior: theory, research & practice.

[27] Berge JM (2009). A review of familial correlates of child and adolescent obesity: what has the 21^st^ century taught us so far?. Int J Adolesc Med Health.

[28] Carlson JA, Saelens BE, Kerr J, Schipperijn J, Conway T, Frank LD (2015). Association between neighborhood walkability and GPS-measured walking, bicycling and vehicle time in adolescents. Health Place.

[29] Pate RR, Mitchell JA, Byun W, Dowda M (2011). Sedentary behaviour in youth. Br J Sports Med.

[30] Laska MN, Hearst MO, Forsyth A, Pasch KE, Lytle L (2010). Neighbourhood food environments: are they associated with adolescent dietary intake, food purchases and weight status?. Public Health Nutr.

[31] van der Horst K, Oenema A, Ferreira I, Wendel-Voss W, Giskes K, van Lenthe F (2007). A systematic review of environmental correlates of obesity-related dietary behaviors in youth. Health Educ Res.

[32] He G, Huang WY, Wong S (2014). Physical activity research in Hong Kong from 1987 to 2012: evidence on children and adolescents. Asia Pac J Public Health.

[33] Ho SY, Wong BY, Lo WZ, Mak KK, Thomas GN, Lam TH (2010). Neighbourhood food environment and dietary intakes in adolescents: sex and perceived family affluence as moderators. Int J Pediatr Obes.

[34] Cerin E, Sit CH, Huang YJ, Barnett A, Macfarlane DJ, Wong SS (2014). Repeatability of self-report measures of physical activity, sedentary and travel behaviour in Hong Kong adolescents for the iHealt(H) and IPEN - adolescent studies. BMC Pediatr.

[35] Kerr J, Sallis JF, Owen N, De Bourdeaudhuij I, Cerin E, Sugiyama T (2013). Advancing science and policy through a coordinated international study of physical activity and built environments: IPEN adult methods. J Phys Act Health.

[36] Shoukri MM, Asyali MH, Donner A (2004). Sample size requirements for the design of reliability study: review and new results. Stat Methods Med Res.

[37] Joe L, Carlson JL. Active Where? Individual item reliability report**,** June, 2010. 2010. http://activelivingresearch.org/sites/default/files/AW_item_reliability_overview.pdf. Accessed 10 Apr 2017.

[38] Joe L, Carlson JL, Sallis JF. Active Where? Individual item reliability statistics adolescent survey. 2010. http://activelivingresearch.org/sites/default/files/AW_item_reliability_Adolescent.pdf. Accessed 10 Apr 2017.

[39] World Health Organization. Process of translation and adaptation of instruments. 2009. http://www.who.int/substance_abuse/research_tools/translation/en. Accessed 10 Apr 2017.

[40] Hagler AS, Norman GJ, Radick LR, Calfas KJ, Sallis JF (2005). Comparability and reliability of paper- and computer-based measures of psychosocial constructs for adolescent fruit and vegetable and dietary fat intake. J Am Diet Assoc.

[41] Kerr J, Rosenberg D, Sallis JF, Saelens BE, Frank LD, Conway TL (2006). Active commuting to school: associations with environment and parental concerns. Med Sci Sports Exerc.

[42] Norman GJ, Sallis JF, Gaskins R (2005). Comparability and reliability of paper- and computer-based measures of psychosocial constructs for adolescent physical activity and sedentary behaviors. Res Q Exerc Sport.

[43] Rosenberg D, Ding D, Sallis JF, Kerr J, Norman GJ, Durant N (2009). Neighborhood environment Walkability scale for youth (NEWS-Y): reliability and relationship with physical activity. Prev Med.

[44] Salmon J, Timperio A, Telford A, Carver A, Crawford D (2005). Association of family environment with children’s television viewing and with low level of physical activity. Obes Res.

[45] Portney LG, Watkins MP (2009). Foundations of Clinical research: applications to practice.

[46] Dassa C, Lambert J, Blais R, Potvin D, Gauthie N (1997). Effects of a neutral answer choice on the reliability and validity of attitude and opinion items. Can J Program Eval.

[47] Chung S. Average home size and accommodation value. Real Estate Tech. 1999. http://www.real-estate-tech.com/articles/Ret0799d1.pdf. Accessed 10 Apr 2017.

[48] Barnett A, Cerin E, Zhang CJ, Sit CH, Johnston JM, Cheung MM (2016). Associations between the neighbourhood environment characteristics and physical activity in older adults with specific types of chronic conditions: the ALECS cross-sectional study. Int J Behav Nutr Phys Act.

[49] Cerin E, Macfarlane D, Sit HP, Ho SY, Johnston JM, Chou KL (2013). Effects of the built environment on walking among Hong Kong elders. Hong Kong Med J.

[50] Kerr J, Emond JA, Badland H, Reis R, Sarmiento O, Carlson J (2016). Perceived neighborhood environmental attributes associated with walking and cycling for transport among adult residents of 17 cities in 12 countries: the IPEN study. Environ Health Perspec.

[51] Cortina JM (1993). What is coefficient alpha? An examination of theory and applications. J Appl Psychol.

[52] Kroll C, Keller R, Scholz U, Perren S (2011). Evaluating decisional balance construct of the Thranstheoretical model: are two dimensions of pros and cons really enough?. Int J Public Health.

[53] Cerin E, Conway TL, Cain KL, Kerr J, De Bourdeaudhij I, Owen N (2013). Sharing good NEWS across the world: developing comparable scores across 12 countries for the neighborhood environment Walkability scale (NEWS). BMC Public Health.

[54] Cerin E, Saelens BE, Sallis JF, Frank LD (2006). Neighborhood environment Walkability scale: validity and development of a short form. Med Sci Sports Exerc.

[55] Cerin E, Leslie E, Owen N, Bauman A (2008). An Australian version of the neighborhood environment Walkability scale: construct and factorial validity. Meas Phys Ed Exerc Sci.

[56] Cerin E, Sit CH, Cheung MC, Ho SY, Lee LC, Chan WM (2010). Reliable and valid NEWS for Chinese seniors: measuring perceived neighborhood attributes related to walking. Int J Behav Nutr Phys Act.

[57] Cerin E, Conway TL, Saelens BE, Frank LD, Sallis JF (2009). Cross-validation of the factorial structure of the neighborhood environment Walkability scale (NEWS) and its abbreviated form (NEWS-A). Int J Behav Nutr Phys Act.

