# Supplementary material for: Reliability of self-report measures of correlates of obesity-related behaviours in Hong Kong adolescents for the iHealt(H) and IPEN adolescent studies
Source: Arch Public Health. 2017 Sep 25;75:38. doi: 10.1186/s13690-017-0209-5 (PMC5611613; doi:10.1186/s13690-017-0209-5)
Supplement: Additional file 1: — Measures of correlates of obesity-related behaviours used in the iHealt(H) and IPEN Adolescent studies(DOCX 60 kb) [file 13690_2017_209_MOESM1_ESM.docx]

**Measures of correlates of obesity-related behaviours used in the iHealt(H) and IPEN Adolescent studies**

**Correlates of Dietary Behaviours - iHealth(H) study**

**Individual Correlates**

***1. Pros for Eating Fruits and Vegetables***

| Please circle the answer that best applies to you when deciding whether or not to eat 5 servings of fruits and vegetables per day? Think about your eating habits over the PAST YEAR. | | | | | |
| --- | --- | --- | --- | --- | --- |
|  | Strongly disagree | Somewhat disagree |  | Somewhat agree | Strongly agree |
| 1. I would have more energy if I ate fruits & vegetables. | 1 | 2 |  | 3 | 4 |
| 2. I would be doing something good for my body if I ate fruits & vegetables. | 1 | 2 |  | 3 | 4 |
| 3. I would feel healthier if I ate fruits & vegetables. | 1 | 2 |  | 3 | 4 |
| 4. My parents would be pleased if I ate fruits & vegetables. | 1 | 2 |  | 3 | 4 |
| 5. Eating fruits & vegetables would be a great way to start the day. | 1 | 2 |  | 3 | 4 |

***2. Cons for Eating Fruits and Vegetables***

| Please circle the answer that best applies to you when deciding whether or not to eat 5 servings of fruits and vegetables per day? Think about your eating habits over the PAST YEAR. | | | | | |
| --- | --- | --- | --- | --- | --- |
|  | Strongly disagree | Somewhat disagree |  | Somewhat agree | Strongly agree |
| 1. I would feel embarrassed if other kids saw me eating fruits & vegetables. | 1 | 2 |  | 3 | 4 |
| 2. I would need too much help from my parents to eat fruits & vegetables. | 1 | 2 |  | 3 | 4 |
| 3. It takes too much time to cut up fruits & vegetables. | 1 | 2 |  | 3 | 4 |
| 4. Fruits & vegetables are too difficult to prepare. | 1 | 2 |  | 3 | 4 |

***3. Pros for Eating High-Fat Foods***

| Please circle the answer that best applies to you when deciding whether or not to eat high-fat foods. Examples of high-fat foods are burgers, pizzas, fries, chips, sweet snacks, deep fried foods, fat pork, chicken skin, etc. Think about your eating habits over the PAST YEAR. | | | | | |
| --- | --- | --- | --- | --- | --- |
|  | Strongly disagree | Somewhat disagree |  | Somewhat agree | Strongly agree |
| 1. Eating my favourite high-fat foods is a quick way to satisfy my hunger. | 1 | 2 |  | 3 | 4 |
| 2. I am happier and more fun to be around when I’m free to eat high-fat foods. | 1 | 2 |  | 3 | 4 |
| 3. My family and friends like me better when I am happy and eating high-fat foods rather than miserable and watching what I eat. | 1 | 2 |  | 3 | 4 |
| 4. I feel good when I’m eating the high- fat foods I enjoy. | 1 | 2 |  | 3 | 4 |

***4. Cons for Eating High-Fat Foods***

| Please circle the answer that best applies to you when deciding whether or not to eat high-fat foods. Examples of high-fat foods are burgers, pizzas, fries, chips, sweet snacks, deep fried foods, fat pork, chicken skin, etc. Think about your eating habits over the PAST YEAR. | | | | | |
| --- | --- | --- | --- | --- | --- |
|  | Strongly disagree | Somewhat disagree |  | Somewhat agree | Strongly agree |
| 1. It bothers other people when I eat a lot of high-fat foods. | 1 | 2 |  | 3 | 4 |
| 2. People close to me disapprove of my eating foods that are too high in fat. | 1 | 2 |  | 3 | 4 |
| 3. Eating high-fat foods now can mean health problems for me in the future. | 1 | 2 |  | 3 | 4 |

***5. Pros for drinking sugar-sweetened beverages***

| Please circle the answer that best applies to you when deciding whether or not to drink sugar-sweetened beverages (soda, sports drinks). Think about your eating habits over the PAST YEAR. | | | | | |
| --- | --- | --- | --- | --- | --- |
|  | Strongly disagree | Somewhat disagree |  | Somewhat agree | Strongly agree |
| 1. I think it is good to drink a lot of sugar-sweetened beverages. | 1 | 2 |  | 3 | 4 |
| 2. I think it is pleasant to drink a lot of sugar-sweetened beverages. | 1 | 2 |  | 3 | 4 |
| 3. Drinking fewer sugar-sweetened beverages seems difficult to me. | 1 | 2 |  | 3 | 4 |

***6. Self-efficacy for eating fruits and vegetables***

| There are many things that can get in the way of eating fruits & vegetables. HOW SURE are you that you can do the following in each situation? Please answer ALL questions. | | | | | |
| --- | --- | --- | --- | --- | --- |
|  | I’m sure  I can’t |  |  |  | I’m sure I can |
| 1. Eat 5 servings of fruits & vegetables every day | 1 | 2 | 3 | 4 | 5 |
| 2. Ask someone in my family to buy my favourite fruit or vegetable | 1 | 2 | 3 | 4 | 5 |
| 3. Ask for fruits & vegetables with my lunch | 1 | 2 | 3 | 4 | 5 |
| 4. Ask someone in my family to include fruits or vegetables with dinner | 1 | 2 | 3 | 4 | 5 |
| 5. Eat fruits & vegetables when eating out at a restaurant | 1 | 2 | 3 | 4 | 5 |

***7. Self-efficacy for eating low-fat foods***

| There are many things that can get in the way of choosing to eat a diet in low-fat foods. Examples of low-fat foods are salads, lean meat (e.g., chicken, fish), etc. HOW SURE are you that you can do the following in each situation? Please answer ALL questions. | | | | | |
| --- | --- | --- | --- | --- | --- |
|  | I’m sure I can’t |  |  |  | I’m sure  I can |
| 1. Ask someone in my family to buy low- fat foods at the grocery store | 1 | 2 | 3 | 4 | 5 |
| 2. Choose low-fat foods during those times when I feel depressed about something | 1 | 2 | 3 | 4 | 5 |
| 3. Choose low-fat foods even when it has been a while since I had a high-fat snack or meal | 1 | 2 | 3 | 4 | 5 |
| 4. Choose low-fat items like grilled/steamed/ barbequed chicken instead of a cheeseburger at a restaurant | 1 | 2 | 3 | 4 | 5 |
| 5. Choose low-fat foods when others around me are eating high-fat foods | 1 | 2 | 3 | 4 | 5 |
| 6. Eat low-fat snacks like seaweed or popcorn without butter instead of high-fat snacks | 1 | 2 | 3 | 4 | 5 |
| 7. Choose low-fat foods when I am craving high-fat food | 1 | 2 | 3 | 4 | 5 |
| 8. Ask someone in my family to prepare meals that are low in fat | 1 | 2 | 3 | 4 | 5 |

***8. Self-efficacy for reducing sugar-sweetened beverage intake***

| There are many things that can get in the way of choosing not to drink sugar-sweetened beverages (soda, sports dink). HOW SURE are you that you can do the following in each situation? Please answer ALL questions. | | | | | |
| --- | --- | --- | --- | --- | --- |
|  | I’m sure I can’t |  |  |  | I’m sure I can |
| 1. Do you think you are able to drink fewer sugar-sweetened beverages? | 1 | 2 | 3 | 4 | 5 |
| 2. How sure are you that you can drink no more than one 330ml can of sugar-sweetened beverage a day? | 1 | 2 | 3 | 4 | 5 |

***9. Enjoyment of fruits and vegetables***

I enjoy eating fruits and vegetables.

| 1  Strongly Disagree | 2  Somewhat Disagree | 3  Neutral | 4  Somewhat Agree | 5  Strongly Agree |
| --- | --- | --- | --- | --- |

***10. Enjoyment of high-fat foods***

Examples of high-fat foods are burgers, pizzas, fries, chips, sweet snacks, deep fried foods, fat pork, chicken skin, etc.

I enjoy eating high-fat foods.

| 1  Strongly Disagree | 2  Somewhat Disagree | 3  Neutral | 4  Somewhat Agree | 5  Strongly Agree |
| --- | --- | --- | --- | --- |

***11. Enjoyment of sugar-sweetened beverages***

I enjoy drinking sugar-sweetened beverages (soda, sports drink).

| 1  Strongly Disagree | 2  Somewhat Disagree | 3  Neutral | 4  Somewhat Agree | 5  Strongly Agree |
| --- | --- | --- | --- | --- |

**Social Correlates**

***1. Social support from adults for eating fruits and vegetables***

|  | Strongly disagree | Somewhat disagree | Somewhat agree | Strongly agree |
| --- | --- | --- | --- | --- |
| 1. My parents think that I should eat fruits and vegetables. | 1 | 2 | 3 | 4 |
| 2. My parents eat fruits and vegetables. | 1 | 2 | 3 | 4 |
| 3. My parents encourage me to eat fruits and vegetables. | 1 | 2 | 3 | 4 |

***2. Social support from peers for eating fruits and vegetables***

|  | Strongly disagree | Somewhat disagree | Somewhat agree | Strongly agree |
| --- | --- | --- | --- | --- |
| 1. My friends think that I should eat fruits and vegetables. | 1 | 2 | 3 | 4 |
| 2. My friends eat fruits and vegetables. | 1 | 2 | 3 | 4 |
| 3. My friends encourage me to eat fruits and vegetables. | 1 | 2 | 3 | 4 |

***3. Social support from adults for eating less high-fat foods***

|  | Strongly disagree | Somewhat disagree | Somewhat agree | Strongly agree |
| --- | --- | --- | --- | --- |
| 1. My parents think that I should eat less high-fat foods. | 1 | 2 | 3 | 4 |
| 2. My parents avoid eating high-fat foods. | 1 | 2 | 3 | 4 |
| 3. My parents encourage me to avoid eating high-fat foods. | 1 | 2 | 3 | 4 |

***4. Social support from peers for eating less high-fat foods***

|  | Strongly disagree | Somewhat disagree | Somewhat agree | Strongly agree |
| --- | --- | --- | --- | --- |
| 1. My friends think that I should eat less high-fat foods. | 1 | 2 | 3 | 4 |
| 2. My friends avoid eating high-fat foods. | 1 | 2 | 3 | 4 |
| 3. My friends encourage me to avoid eating high-fat foods. | 1 | 2 | 3 | 4 |

***5. Social support from adults for drinking sugar-sweetened beverages***

|  | Strongly disagree | Somewhat disagree | Somewhat agree | Strongly agree |
| --- | --- | --- | --- | --- |
| 1. My parents think that I should drink sugar-sweetened beverages. | 1 | 2 | 3 | 4 |
| 2. My parents drink sugar-sweetened beverages. | 1 | 2 | 3 | 4 |
| 3. My parents encourage me to drink sugar-sweetened beverages. | 1 | 2 | 3 | 4 |

***6. Social support from peers for drinking sugar-sweetened beverages***

|  | Strongly disagree | Somewhat disagree | Somewhat agree | Strongly agree |
| --- | --- | --- | --- | --- |
| 1. My friends think that I should drink sugar-sweetened beverages. | 1 | 2 | 3 | 4 |
| 2. My friends drink sugar-sweetened beverages. | 1 | 2 | 3 | 4 |
| 3. My friends encourage me to drink sugar-sweetened beverages. | 1 | 2 | 3 | 4 |

**Environmental Correlates**

***1. School food environment (unhealthy)***

| 1. Are there food vending machines at your school? | 1. Yes | 0. No | |
| --- | --- | --- | --- |
|  |  |  | |
| 2. Are there drink vending machines at your school? | 1. Yes | 0. No | |
|  |  |  | |
| 3. Is there usually a salad bar at your school? | 1. Yes | 0. No | |
|  |  | |  |
| 4. Can you buy brand named fast foods (e.g., Pizza Hut, MacDonald’s) at your school? | 1. Yes | | 0. No |

**Correlates of Physical Activity – iHealt(H) and IPEN Adolescent studies**

**Individual Correlates**

***1. Perceived barriers to active transport to/from school***

| It is difficult for me to walk or bike to my school because… | | | | |
| --- | --- | --- | --- | --- |
|  | Strongly disagree | Somewhat disagree | Somewhat agree | Strongly agree |
| 1. There are no sidewalks or bike lanes | 1 | 2 | 3 | 4 |
| 2. The route is boring | 1 | 2 | 3 | 4 |
| 3. The route does not have good lighting | 1 | 2 | 3 | 4 |
| 4. There are one or more dangerous crossings | 1 | 2 | 3 | 4 |
| 5. I get too hot and sweaty | 1 | 2 | 3 | 4 |
| 6. No other teens walk or bike | 1 | 2 | 3 | 4 |
| 7. It’s not considered cool to walk or bike | 1 | 2 | 3 | 4 |
| 8. I have too much stuff to carry | 1 | 2 | 3 | 4 |
| 9. It‘s easier to drive or get driven there | 1 | 2 | 3 | 4 |
| 10. It involves too much planning ahead | 1 | 2 | 3 | 4 |
| 11. There is nowhere to leave a bike safely | 1 | 2 | 3 | 4 |
| 12. There are stray dogs | 1 | 2 | 3 | 4 |
| 13. It is too far | 1 | 2 | 3 | 4 |
| 14. I would have to walk/bike through places that were unsafe because of crime or things sometimes related to crime (e.g., vandalism, graffiti, people drinking alcohol in public places) | 1 | 2 | 3 | 4 |
| 15. I don’t enjoy walking or biking to school | 1 | 2 | 3 | 4 |
| 16. There are too many hills | 1 | 2 | 3 | 4 |
| 17. There is too much traffic | 1 | 2 | 3 | 4 |
| 18. I am tired | 1 | 2 | 3 | 4 |
| 19. I have a tight schedule (no time) | 1 | 2 | 3 | 4 |

***2. Perceived barriers to active transport to/from closest park***

| It is difficult for me to walk or bike to the closest **local park** because… | | | | | |
| --- | --- | --- | --- | --- | --- |
|  | Strongly disagree | Somewhat disagree | Somewhat agree | Strongly agree | |
| 1. There are no sidewalks or bike lanes | 1 | 2 | 3 | 4 | |
| 2. The route is boring | 1 | 2 | 3 | 4 | |
| 3. The route does not have good lighting | 1 | 2 | 3 | 4 | |
| 4. There are one or more dangerous crossings | 1 | 2 | 3 | 4 | |
| 5. I get too hot and sweaty | 1 | 2 | 3 | 4 | |
| 6. No other teens walk or bike | 1 | 2 | 3 | 4 | |
| 7. It’s not considered cool to walk or bike | 1 | 2 | 3 | 4 | |
| 8. I have too much stuff to carry | 1 | 2 | 3 | 4 | |
| 9. It is easier to drive there on the way to something else | 1 | 2 | 3 | 4 | |
| 10. It involves too much planning ahead | 1 | 2 | 3 | 4 | |
| 11. There is nowhere to leave a bike safely | 1 | 2 | 3 | 4 | |
| 12. There are stray dogs | 1 | 2 | 3 | 4 | |
| 13. It is too far | 1 | 2 | 3 | 4 | |
| 14. I don’t enjoy walking or biking to the park | 1 | 2 | 3 | 4 | |
| 15. I would have to walk/bike through places that were unsafe because of crime or things sometimes related to crime (e.g., vandalism, graffiti, people drinking alcohol in public places). | 1 | 2 | 3 | 4 | |
| 16. There are too many hills | 1 | 2 | 3 | | 4 |
| 17. There is too much traffic | 1 | 2 | 3 | | 4 |

***3. Perceived barriers to physical activity in the neighbourhood***

| It’s difficult for me to be active in the local park or streets/neighbourhood near our home because… | | | | |
| --- | --- | --- | --- | --- |
|  | Strongly disagree | Somewhat disagree | Somewhat agree | Strongly agree |
| 1. There is no choice of activities | 1 | 2 | 3 | 4 |
| 2. There is no equipment (basketball hoop, etc.) | 1 | 2 | 3 | 4 |
| 3. There is no adult supervision | 1 | 2 | 3 | 4 |
| 4. There are no other teens there | 1 | 2 | 3 | 4 |
| 5. It’s not safe because of crime (strangers, gangs, drugs) | 1 | 2 | 3 | 4 |
| 6. It is not safe because of traffic | 1 | 2 | 3 | 4 |
| 7. It does not have good lighting | 1 | 2 | 3 | 4 |
| 8. I have been a victim of crime in my neighbourhood | 1 | 2 | 3 | 4 |
| 9. Someone I know has been a victim of crime in my neighbourhood | 1 | 2 | 3 | 4 |

***4. Pros for engagement in physical activity***

| Please circle the answer that best applies to you when deciding whether or not to do physical activity. | | | | | | |
| --- | --- | --- | --- | --- | --- | --- |
|  |  | Strongly disagree | Somewhat disagree |  | Somewhat agree | Strongly agree |
| 1. | Physical activity would help me stay fit. | 1 | 2 |  | 3 | 4 |
| 2. | My parents would be happy if I did physical activity. | 1 | 2 |  | 3 | 4 |
| 3. | I would feel better about myself if I did physical activity. | 1 | 2 |  | 3 | 4 |
| 4. | I would have fun doing physical activity or playing sports with my friends. | 1 | 2 |  | 3 | 4 |
| 5. | I would have more energy if I did physical activity. | 1 | 2 |  | 3 | 4 |

***5. Cons for engagement in physical activity***

| Please circle the answer that best applies to you when deciding whether or not to do physical activity. | | | | | | |
| --- | --- | --- | --- | --- | --- | --- |
|  |  | Strongly disagree | Somewhat disagree |  | Somewhat agree | Strongly agree |
| 1. | I would feel embarrassed if people saw me doing physical activity. | 1 | 2 |  | 3 | 4 |
| 2. | There is too much I would have to learn to do physical activity. | 1 | 2 |  | 3 | 4 |
| 3. | I would need too much help from my parents to do physical activity. | 1 | 2 |  | 3 | 4 |
| 4. | I do not like the way physical activity and exercise makes me feel. | 1 | 2 |  | 3 | 4 |
| 5. | Physical activity takes time away from being with my friends. | 1 | 2 |  | 3 | 4 |

***6. Self-efficacy for physical activity***

| There are many things that can get in the way of physical activity. HOW SURE are you that you can do physical activity in each situation? Please answer ALL questions**.** Think about the PAST YEAR. | | | | | | |
| --- | --- | --- | --- | --- | --- | --- |
|  |  | I’m sure I can’t |  |  |  | I’m sure  I can |
| 1. | Do physical activity even when you feel sad or stressed | 1 | 2 | 3 | 4 | 5 |
| 2. | Set aside time for physical activity on most days of the week | 1 | 2 | 3 | 4 | 5 |
| 3. | Do physical activity even when your family or friends want you to do something else | 1 | 2 | 3 | 4 | 5 |
| 4. | Get up early, even on weekends, to do physical activity | 1 | 2 | 3 | 4 | 5 |
| 5. | Do physical activity even when you have a lot of homework | 1 | 2 | 3 | 4 | 5 |
| 6. | Do physical activity even when it is raining or really hot outside | 1 | 2 | 3 | 4 | 5 |

***7. Enjoyment of physical activity***

I enjoy doing physical activity.

| 1  Strongly Disagree | 2  Somewhat Disagree | 3  Neutral | 4  Somewhat Agree | 5  Strongly Agree |
| --- | --- | --- | --- | --- |

**Social Correlates**

***1. Social support from adults for physical activity***

| During a typical week, how often does an **adult in your household:** | | | | | |
| --- | --- | --- | --- | --- | --- |
|  | Never | Rarely | Sometimes | Often | Very Often |
| 1. Encourage you to do sports or physical activity? | 0 | 1 | 2 | 3 | 4 |
| 2. Provide transportation to a place where you can do physical activity or play sports? | 0 | 1 | 2 | 3 | 4 |
| 3. Do physical activity or play sports with you? | 0 | 1 | 2 | 3 | 4 |

***2. Social support from peers for physical activity***

| During a typical week how often do your **brothers/sisters or friends:** | | | | | |
| --- | --- | --- | --- | --- | --- |
|  | Never | Rarely | Sometimes | Often | Very Often |
| 1. Do physical activity or play sports with you? | 0 | 1 | 2 | 3 | 4 |
| 2. Ask you to walk or bike to school or to a friend’s house? | 0 | 1 | 2 | 3 | 4 |

***3. Parental rules about physical activity***

| Does your parent or guardian have the following rules, whether they remind you often or not? Please circle an answer for each rule. | | |
| --- | --- | --- |
| 1. Stay close to or within sight of your home/parent | Yes | No |
| 2. Come in before dark | Yes | No |
| 3. Do not go to places alone | Yes | No |
| 4. Stay in the neighbourhood | Yes | No |
| 5. Do not ride bike on street | Yes | No |
| 6. Carry a cell phone or 2-way radio | Yes | No |
| 7. Do homework before going out | Yes | No |
| 8. Watch out for cars | Yes | No |
| 9. Check in frequently | Yes | No |
| 10. Stay on paths, trails or sidewalk | Yes | No |
| 11. Do not cross busy streets | Yes | No |
| 12. Wear hat and/or sunscreen in summer | Yes | No |
| 13. Do not fight with other kids | Yes | No |
| 14. Do not disrespect others (particularly adults) | Yes | No |

**Environmental Correlates**

***1. School physical activity equipment***

| Do you have any of these at your school? | | | |
| --- | --- | --- | --- |
| 1. | Basketball hoops | Yes | No |
| 2. | Soccer goal posts | Yes | No |
| 3. | Balls | Yes | No |
| 4. | Running/walking track | Yes | No |
| 5. | Weight-lifting machines | Yes | No |
| 6. | Indoor exercise machines such as treadmills/stair climbers | Yes | No |

***2. Physical activity equipment at home***

| Do you have any of these at home (or in a common apartment area)? |  |  |
| --- | --- | --- |
| 1. Bike | Yes | No |
| 2. Basketball hoop | Yes | No |
| 3. Jump rope | Yes | No |
| 4. Active video games (like Dance Dance Revolution, Wii, etc.) | Yes | No |
| 5. Sports equipment (like balls, racquets, bats, sticks) | Yes | No |
| 6. Swimming pool | Yes | No |
| 7. Rollerblades, skateboard, scooter | Yes | No |
| 8. Home aerobic equipment (like treadmill, stationary bike, workout videos) | Yes | No |
| 9. Weight-lifting equipment (like free-weights, weight machines) | Yes | No |
| 10. Water or snow equipment (like skis, kayak, snowboard) | Yes | No |

***3. Perceived neighbourhood traffic safety***

| Please circle the answer that best applies to you and your **local neighbourhood,** which means within a 10-15 minute walk from your home. Please circle one response for each item. | | | | |
| --- | --- | --- | --- | --- |
|  | Strongly disagree | Somewhat disagree | Somewhat agree | Strongly agree |
| 1. There is so much traffic along nearby streets that it makes it difficult or unpleasant for me to walk (alone or with someone) in my neighbourhood. | 1 | 2 | 3 | 4 |
| 2. The speed of traffic on most streets is usually slow (40 km or less). | 1 | 2 | 3 | 4 |
| 3. Most drivers go faster than the posted speed limits. | 1 | 2 | 3 | 4 |
| 4. Streets have good lighting at night. | 1 | 2 | 3 | 4 |
| 5. There are crosswalks and signals on busy streets. | 1 | 2 | 3 | 4 |
| 6. I feel safe crossing the streets in my neighbourhood. | 1 | 2 | 3 | 4 |

***4. Perceived neighbourhood crime safety***

| Please circle the answer that best applies to you and your **local neighbourhood,** which means within a 10-15 minute walk from your home. Please circle one response for each item. | | | | |
| --- | --- | --- | --- | --- |
|  | Strongly disagree | Somewhat disagree | Somewhat agree | Strongly agree |
| 1. I am worried about being in a local/nearby park because I am afraid of being taken or hurt by a stranger. | 1 | 2 | 3 | 4 |
| 2. Walkers and bikers can be easily seen by people in their homes. | 1 | 2 | 3 | 4 |
| 3. There is a high crime rate. | 1 | 2 | 3 | 4 |
| 4. I am worried about being or walking alone or with friends in my neighbourhood and local streets because I am afraid of being taken or hurt by a stranger. | 1 | 2 | 3 | 4 |
| 5. I am worried about being outside alone around my home because I am afraid of being taken or hurt by a stranger. | 1 | 2 | 3 | 4 |
| 6. I’m afraid of being taken or hurt by a known “bad” person in my neighbourhood. | 1 | 2 | 3 | 4 |
| 7. The crime rate in my neighbourhood makes it unsafe to go on walks alone or with someone at night | 1 | 2 | 3 | 4 |
| 8. I am worried about being outside with a friend around my home because I am afraid of being taken or hurt by a stranger on local streets. | 1 | 2 | 3 | 4 |

***5. Physical activity friendly school policy***

1. How often does your school have supervised physical activities after school?

0 1 2 3 4

Never Rarely Sometimes Frequently Always

2. How often does your school allow students to use play areas or fields after school?

0 1 2 3 4

Never Rarely Sometimes Frequently Always

**Correlates of Sedentary Behaviour – iHealt(H) and IPEN adolescent studies**

**Individual Correlates**

***1. Pros for engagement in sedentary behaviour***

| Please circle the answer that best applies to you when deciding whether or not to do sedentary activities? | | | | | | | |
| --- | --- | --- | --- | --- | --- | --- | --- |
|  |  | Strongly disagree | Somewhat disagree |  | Somewhat agree | Strongly agree |  |
| 1. | I enjoy playing computer/video games for many hours at a time. | 1 | 2 |  | 3 | 4 |  |
| 2. | Watching TV or playing computer/video games is my way to escape from the world. | 1 | 2 |  | 3 | 4 |  |
| 3. | I feel good about myself when I do well at my favourite computer/video games. | 1 | 2 |  | 3 | 4 |  |
| 4. | Watching TV is one of my favourite forms of entertainment. | 1 | 2 |  | 3 | 4 |  |
| 5. | I find sitting and watching TV very relaxing. | 1 | 2 |  | 3 | 4 |  |
| 6. | My friends would be disappointed if I tried to spend less time chatting with them (e.g., talking on the phone, emailing, texting). | 1 | 2 |  | 3 | 4 |  |

***2. Cons for engagement in sedentary behaviour***

| Please circle the answer that best applies to you when deciding whether or not to do sedentary activities? | | | | | | |
| --- | --- | --- | --- | --- | --- | --- |
|  |  | Strongly disagree | Somewhat disagree |  | Somewhat agree | Strongly agree |
| 1. | I think TV and computer/video games are boring. | 1 | 2 |  | 3 | 4 |
| 2. | Watching TV takes time away from doing other, more important things. | 1 | 2 |  | 3 | 4 |
| 3. | I would feel lazy and sluggish if I sat and watched TV for many hours. | 1 | 2 |  | 3 | 4 |
| 4. | I see too many commercials when I watch a lot of TV. | 1 | 2 |  | 3 | 4 |
| 5. | My parents would be pleased if I spent less time playing computer/video games. | 1 | 2 |  | 3 | 4 |
| 6. | Playing computer/video games sometimes hurts my eyes and gives me a headache. | 1 | 2 |  | 3 | 4 |

***3. Self-efficacy for reducing sedentary behaviour***

| There are many situations where you can reduce the amount of time that you spend on sedentary habits. HOW SURE are you that you can do the following in each situation? Please answer ALL questions. | | | | | | |
| --- | --- | --- | --- | --- | --- | --- |
|  |  | I’m sure  I can’t |  |  |  | I’m sure  I can |
| 1. | Turn off the TV even when there is a program on you enjoy | 1 | 2 | 3 | 4 | 5 |
| 2. | Limit your online computer time (e.g., emailing, browsing) to 1 hour per day | 1 | 2 | 3 | 4 | 5 |
| 3. | Leave the room where the TV is on, even if others are watching it | 1 | 2 | 3 | 4 | 5 |
| 4. | Plan ahead of time what TV shows you will watch during the week | 1 | 2 | 3 | 4 | 5 |
| 5. | Instead of just sitting listening to music, listen while you are being active (e.g., walking or dancing) | 1 | 2 | 3 | 4 | 5 |
| 6. | Set limits on how long you plan to talk on the telephone or text message with friends | 1 | 2 | 3 | 4 | 5 |
| 7. | Limit TV, video and computer games to only 2 hours per day | 1 | 2 | 3 | 4 | 5 |

***4. Enjoyment of sedentary behaviour***

I enjoy doing sedentary activities like watching TV or playing computer/video games.

| 1  Strongly Disagree | 2  Somewhat Disagree | 3  Neutral | 4  Somewhat Agree | 5  Strongly Agree |
| --- | --- | --- | --- | --- |

**Social Correlates**

***1. Social support from adults for sedentary behaviour***

| During a typical week, how often do you sit and watch TV or play electronic games (do not include time in exercise games like Wii or Dance Dance Revolution) with… | | | | | |
| --- | --- | --- | --- | --- | --- |
|  | Never | 1-2 days | 3-4 days | 5-6 days | Every day |
| A parent/guardian/caregiver | 0 | 1 | 2 | 3 | 4 |

***2. Social support from peers for sedentary behaviour***

| During a typical week, how often do you sit and watch TV or play electronic games (do not include time in exercise games like Wii or Dance Dance Revolution) with… | | | | | |
| --- | --- | --- | --- | --- | --- |
|  | Never | 1-2 days | 3-4 days | 5-6 days | Every day |
| 1. Brothers/sisters   (if no brothers or sisters, circle ‘Never’) | 0 | 1 | 2 | 3 | 4 |
| 2. Friends | 0 | 1 | 2 | 3 | 4 |

***3. Parental rules about sedentary behaviour***

| Does your parent or guardian have the following rules, whether they remind you often or not? | | |
| --- | --- | --- |
| 1. No TV/DVD/computer before homework | Yes | No |
| 2. Less than 2 hours TV/DVD/computer per day | Yes | No |
| 3. No internet use without permission | Yes | No |

**Environmental Correlates**

***1. Screen media in bedroom***

| Please indicate whether the following is in your bedroom. | | |
| --- | --- | --- |
| 1. TV | Yes | No |
| 2. VCR or DVD player | Yes | No |
| 3. Music player (radio, CD or tape player, stereo) | Yes | No |
| 4. Computer | Yes | No |
| 5. Video game system (non-hand held—Playstation, Xbox, etc.) | Yes | No |
| 6. Internet access | Yes | No |

1. ***Personal electronics***

| Do you have the following items for your own use? | | |
| --- | --- | --- |
| 1. Cell phone or 2-way radio | Yes | No |
| 2. Hand held videogame player (Game Boy, Sony PSP, etc.) | Yes | No |
| 3. Personal stereo (iPod, MP3 player, Discman) | Yes | No |
| 4. Your own website, MySpace or Facebook page | Yes | No |
